# Supplementary material for: Focal Boost in Prostate Cancer Radiotherapy: A Review of Planning Studies and Clinical Trials
Source: Cancers (Basel). 2023 Oct 8;15(19):4888. doi: 10.3390/cancers15194888 (PMC10572027; doi:10.3390/cancers15194888)
Supplement: Supplementary file 1 [file cancers-15-04888-s001.zip › cancers-2590396-supplementary.pdf]

Supplemental Material

Note

"N" represents the reported number of patients/plans who have been focal boosted or included in statistics of dose metrics.

The format of dose metrics reported in "Dose Statistics" is: **reported target volume-[statistics of cohort (sub-statistics)/(unit)]**.

- For example, the reporting format of "**PTVboost-[Median(range)/Gy]**" the reported dose merics of "**Dmedian = 55.3 (49.5~61.8)**" means: for the target volume of "PTVboost", the median of Dmedian in investigated cohort is 55.3 Gy with range of 49.5 to 61.8.

The reported dose metrics included:

- DX: (Maximum) Dose received by X% of the volume
- DXcc: (Maximum) Dose received by X cc of the volume.
- DX%: Percent of isodose received by X% volume of the volume.
- VX: The percentage volume receiving >=XGy
- VX%: Percent of the volume received X% of prescribed dose.

Table S1

Table S1: Summarized characteristics of reviewed planning studies.

Authors, Year	N	Boost Modality	GTV identification modality	Volume definition and margins (mm)		GTV/boost Volume (cc)	Dose and Fractions (fx)			Dose Statistics			
				CTV	PTV		PTV	GTV [BED]	fx	Prostate	GTV	Bladder	Rectum
Cambria et al.,2012 [1]	10	IMPT (2 fields)	mpMRI (T2w+DCE+DWI)	CTV = prostate	PTV = CTV + 5 (3 posteriorly) PTVboost = GTV + 3	[GTV]: Vmean = 1.7 (95%CI: 1~2.7) [PTVboost]: Vmean = 6.2 (95%CI: 3.8~8.6)	PTV = 36.25 Gy	PTVboost = 37.5 Gy [128.2]	5	PTV-[Mean/%]: D98% = 94.8; D95% = 96.8; D50% = 103.4; D2% = 107.6	PTVboost-[Mean/%]: D98% = 98.4; D95% = 98.8; D50% = 101; D2% = 103.9	[Mean/%]: V100% = 1.01; V50% = 8.76	[Mean/%]: V50% = 11.03; V80% = 5.22; V90% = 2.68; V100% = 0.69
		IMPT (5 fields)								PTV-[Mean/%]: D98% = 96.4; D95% = 99.6; D50% = 103.6; D2% = 107.4	PTVboost-[Mean/%]: D98% = 98.7; D95% = 99.0; D50% = 100.7; D2% = 104.0	[Mean/%]: V100% = 0.49; V50% =8.05	[Mean/%]: V50% = 8.81; V80% = 3.8; V90% = 2.17; V100% = 0.4
		VMAT								PTV-[Mean/%]: D98% = 96.4; D95% = 96.8; D50% = 99.8; D2% = 104.7	PTVboost-[Mean/%]: D98% = 96.5; D95% = 97.0; D50% = 99.3; D2% = 102.1	[Mean/%]: V100% = 0.68; V50% =12.41	[Mean/%]: V50% = 18.91; V80% = 6.5; V90% = 3.2; V100% = 0.27
		IMRT								PTV-[Mean/%]: D98% = 94.3; D95% = 96.7; D50% = 100.3; D2% = 102.4	PTVboost-[Mean/%]: D98% = 96.3; D95% = 97.5; D50% = 99.6; D2% = 101.3	[Mean/%]: V100% = 0.66; V50% =11.15	[Mean/%]: V50% = 11.63; V80% = 4.56; V90% = 1.68; V100% = 0.09

Udrescu et al.,2013 [2]	9	IMRT	mpMRI (T2w+DCE)	CTV = prostate	PTV =CTV + 3<br>PTVboost = GTV + 5	[PTVboost]: Vmean = 15.0cc (range:7.9~27.2)	PTV = 32.5 Gy	PTVboost = 40 Gy [143.2]	5	The average PTV coverage by the 95% isodoseline was 98.85% (range 97.8~99.81%)	The average PTVboost coverage by the 95% isodoseline was of 99.36% (range 97.72~99.98%).	[Mean(range)/Gy]: D2,5,10,25 cc = 31.5 (26~34), 26.4 (17.5~32.4), 20.6 (10.2~29.2), 10.9 (2.3~19.5); Dmean = 7.8 (3.2~16.1); Dmax = 37.3 (34.2~41.9).	[Mean(range)/Gy]: D2,5,10,25cc = 35.6 (33.2~38.7), 31.9 (29.8~34.8), 26.6 (23.3~30.7), 14.9 (11.9~16.8); Dmean = 11.2 (9.2~13.4); Dmax = 42 (40.3~43.2).
Y. J. Kim, Yoon, & Kim,2020 [3]	15	CyberKnife	mpMRI (T2w+DWI)	CTV = prostate and proximal SV	PTV was a 2–3 mm expansion of CTV.	Vmedian= 1.3 (range: 0.7–6.6)	Postate = 35 Gy	GTV = 40 Gy [143.2]	5	Prostate-[Mean(SD)/Gy]: D95 = 35.10 (0.81)	GTV-[Mean(SD)/Gy]: D95 = 38.85 (0.80)	[Mean(SD)/Gy]: Dmax = 37.31 (0.83)<br>[Mean(SD)/cc]: D35.7Gy = 0.46 (0.30)	[Mean(SD)/Gy]: Dmax = 34.96 (1.48)<br>[Mean(SD)/cc]: D30.8Gy = 1.00 (1.23)
			mpMRI (T2w) + biopsy					GTV = 45 Gy [175.6]	5	Prostate-[Mean(SD)/Gy]: D95 = D95 = 35.23 (0.68)	GTV-[Mean(SD)/Gy] D95 = 42.89 (1.09)	[Mean(SD)/Gy]: Dmax = 39.13 (2.07)<br>[Mean(SD)/cc]: D35.7Gy =1.17 (1.50)	[Mean(SD)/Gy]: Dmax = 37.15 (3.44)<br>[Mean(SD)/cc]: D30.8Gy =2.14 (2.67)
Tree, Jones, Sohaib, Khoo, & van As,2013 [4]	15	VMAT	mpMRI (T2w+DWI)	CTV = prostate + proximal SV;	PTV = CTV + 5 (3 posteriorly)	[GTV] Vmean = 3.7	PTV = 36.25 Gy	GTV = 47.5 Gy [193.1]	5	NR	[Median/Gy]: D95% = 47.5<br>[mean/Gy]: D95% = 47.4	NR	[Mean/Gy]: D50,20,10,5 = 113.17, 24.37, 30.86, 34.29; D1cc = 36.78
		CyberKnife									[Mean/Gy]: D95% = 47.5<br>[mean/Gy]: D95% = 48.1		[Mean/Gy]: D(0,20,10,5 = 15.52, 27.13, 32.40, 35.32; D1cc = 37.15
		VMAT			PTV = CTV + 8 (5 posteriorly)						[Mean/Gy]: D95% = 46.9		[Mean/Gy]: D50,20,10,5 = 14.59,28.28,34.61,36.63; D1cc = 37.7
Murray et al., 2014 [5]	10	VMAT	mpMRI (DWI+T2w+DCE)	CTV1 = prostate<br>CTV2 = prostate + proximal SV	PTVboost = GTV + 4<br>PTV1 = CTV1 + 6<br>PTV2 = CTV2 + 6	[PTVboost] Vmedian = 3.4 (range:1.5~51.6)	PTV1 = 42.7 Gy	PTVboost = increased in 5% increments starting at 115% of the PTV1 prescription until dose constraints were reached	7	NR	PTVboost-[Median(range)/Gy]: Dmedian = 55.1 (49.6~62.6)	NR	NR
							PTV1 = 42.7 Gy<br>PTV2 = 32.4 Gy				PTVboost-[Median(range)/Gy]: Dmedian = 54.9 (50.1~62.5)		
							PTV1 = 42.7 Gy				PTVboost-[Median(range)/		

							PTV2 = 36.5 Gy				Gy]: Dmedian = 55.3 (49.5~61.8)		
**Ashida et al., 2022 [6]**	14	VMDWAT	mpMRI (DWI+T2w)	CTV = Prostate + proximal SV	PTV = CTV + 8 (6 posteriorly) PTVboost = GTV + 3	[GTV] Vmean = 2.40 (SD: 2.31)	PTV = 54 Gy	PTVboost = 57 Gy [126.9]	15	PTV-[Mean(SD)/Gy]: D95 = 51.25 (0.28); D50 = 54.81 (0.24); D2 = 56.51 (SD: 0.21)	PTVboost-[Mean(SD)/Gy]: D95 = 55.04 (0.76); D50 = 57.07 (0.13); D2 = 58.82 (SD: 0.49)	[Mean(SD)/%]: V30Gy = 26.45 (7.26); V50Gy = 14.99 (4.49)	[Mean(SD)/%]: V30Gy = 39.36 (5.38); V50Gy = 10.44 (2.55)
**Ciabatti et al.,2019 [7]**	12	VMAT (without sexual sparing)	mpMRI (DWI+T2w+DCE)	CTV1 = prostate CTV2 = SV	PTV1 = CTV1 + 5 PTV2 = CTV2 + 5 PTVboost = GTV+ 5	[GTV]: Vmean =0.5 (0.1-6.8) [PTVboost]: Vmean = 7.6 (SD: 6.2)	PTV1 = 67.5 Gy PTV2 = 56.25 Gy	PTVboost = 75 Gy [147.6]	25	PTV1-[Mean(SD)/Gy]: Dmean = 68.9 (1.2); D95 = 66.3 (0.7); D50 = 68.1 (1.2); D2 = 76.2 (1.4)	PTVboost-[Mean(SD)/Gy]: Dmean = 75.7 (1.2); D95 = 73.6 (0.9); D50 = 75.9 (1.2); D2 = 77.6 (1.8)	[Mean(SD)/Gy]: Dmean = 23.8 (7.9); D2 = 68.0 (2.2) [Mean/%]: V80,75,70,65 = 1.5,5.6,8.4,10.9	[Mean(SD)/Gy]: Dmean = 38.1 (7.0); D2 = 68.8 (2.9) [Mean/%]: V80,75,70,65 = 6.1,9.7,13.4,17.2,29.9
		VMAT (with sexual sparing)								PTV1-[Mean(SD)/Gy]: Dmean = 68.8 (0.9); D95 = 65.9 (0.9); D50 = 68.0 (0.8); D2 = 76.4 (1.1)	PTVboost-[Mean(SD)/Gy]: Dmean = 75.8 (0.8); D95 = 73.9 (0.8); D50 = 75.9 (0.8); D2 = 77.7 (1.3)	[Mean(SD)/Gy]: Dmean = 23.2 (8.1); D2 = 67.9 (1.7) [Mean/%]: V80,75,70,65 = 3.9,5.2,5.9,6.6	[Mean(SD)/Gy]: Dmean = 38.1 (7.3); D2 = 68.7 (2.2) [Mean/%]: V80,75,70,65 = 5.4,9.1,12.9,17.2,30.9
**Azzeroni et al., 2012 [8]**	7	IMRT	mpMRI (T1w+T2w+DWI)	CTV = prostate + SV	PTVboost = GTV + 5 PTV = CTV + 8 (10 in cranial-caudal direction)	NR	Biologically optimized	Biologically optimized	28	Median doses were in the range of 69-77 Gy	Median doses were in the range of 94-116 Gy	NR	NR
**Amini, Westerly, Waxweiler, Ryan, & Raben, 2015 [9]**	10	VMAT	Biopsies	CTV1 = involved lobe CTV2 = uninvolved lobe	PTV = CTV1 + 5 (3 posteriorly) + 3 mm lateral margin overlapping the CTV2	NR	Uninvolved lobe: 50.4 Gy	Involved lobe: 70 Gy [126.5]	28	Uninvolved lobe-[Mean/Gy]: Dmean = 55.5 (range: 54.1~56.5)	Involved lobe-[Mean]: Dmean = 72.2 (range:71.6~73.5)	[Mean/Gy]: Dmean = 13.0 (range: 3.1~23.9) [Mean/%]: V70 = 1.69, V60 = 3.79, V50 = 6.96	[Mean/Gy]: Dmean = 21.0 (range: 13.9~27.0) [Mean/%]: V70 = 2.01; V60 = 5.69, V50 = 10.06
**Thomas et al.,2018 [10]**	21	VMAT	PET-CT (68Ga-PSMA)	CTV1 = prostate CTV2 = SV GTV = PET defined boost volume	PTV1 = CTV1 + 7 (4 inferiorly and posteriorly) PTV2 = CTV2 + 7 (4 inferiorly and posteriorly)	NR	PTV1 = 70 Gy PTV2 = 59.2 Gy	GTV = 75.6 Gy [141.4]	28	PTV1-[Mean(SD)/Gy]: Dmean = 74.1(2.9); D1 = 79.9 (1.2) -[Mean(SD)/%]: V95 = 69.6(1.0);	GTV-[Mean(SD)/Gy]: Dmean = 78.0 (0.8); D1 = 81.0 (1.5) -[Mean(SD)/%]: V95 = 75.6% (0.2%);	[Mean/Gy]: Dmean = 38.6 (9.9); D1 =74.5 (1.5)	[Mean/Gy]: Dmean = 27.5 (3.0); D1 =67.8 (4.1)
								GTV = 80 Gy [153.7]		PTV1-[Mean(SD)/Gy]: Dmean = 76.1(1.1); D1 =	GTV-[Mean(SD)/Gy]: Dmean = 83.4 (1.1); D1 = 87.1	[Mean/Gy]: Dmean = 39.9 (10.8); D1 =77.7 (2.2)	[Mean/Gy]: Dmean = 27.1 (3.8); D1 =70.1 (4.3)

										78.3 (1.6)<br>-[Mean(SD)/%]: V95 = 70.9(1.4)	(2.3)<br>-[Mean(SD)/%]: V95 = 80.0% (0%)		
							NA	GTV = 75 Gy [139.8]		PTV1-[Mean(SD)/Gy]: Dmean = 36.9 (11.7); D1 = 85.0 (2.4)<br>-[Mean(SD)/%]: V95 = 3.4% (6.9%)	GTV-[Mean(SD)/Gy]: Dmean = 77.3 (1.2); D1 = 79.3 (1.7)<br>-[Mean(SD)/%]: V95 = 75.0% (0.2%)	[Mean/Gy]: Dmean = 4.6 (3.8); D1 =29.5 (21.8)	[Mean/Gy]: Dmean = 17.7 (3.8); D1 =61.0 (13.7)
							NA	GTV = 95 Gy [199.0]		PTV1-[Mean(SD)/Gy]: Dmean = 43.4 (15.8); D1 = 101.1 (2.7)<br>-[Mean(SD)/%]: V95 = 4.0% (8.7%)	GTV-[Mean(SD)/Gy]: Dmean = 99.4 (1.8); D1 = 103.3 (2.5)<br>-[Mean(SD)/%]: V95 = 95.0% (0.1%)	[Mean/Gy]: Dmean = 4.8 (4.3); D1 =33.7 (28.3)	[Mean/Gy]: Dmean = 17.8 (3.2); D1 =68.8 (16.8)
**Wang et al.,2020 [11]**	36	IMPT	mpMRI (T2WI+ T1WI+DWI)	The plans were robustly optimized with 6 mm setup uncertainty in all directions except for 0 mm laterally,		[GTV]: Vmean = 0.63 (std:0.53, range: 0.09~2.27)	70 Gy (assuming a proton RBE of 1.1)	D95>=140%; D90>=200% (% of prescribed dose to prostate)	28	Prostate-[Mean(SD)/%]: D90% = 100.4 (0.1); V100% = 98.0 (0.1); V110% = 20.4 (8.5)	GTV-[Mean(SD)/%]: D90%,95% = 155.2 (24.6), 147.3 (25.2); V100%,125%,150%,175% = 99.9 (0.3),93.0 (17.2),81.8 (28.5),64.0 (33.9)	[Mean(SD)/Gy]: D15,25,35,50 = 32.0 (21.4),14.9 (16.5),6.8 (10.5),2.0 (4.0)	[Mean(SD)/Gy]: D15,25,35,50 = 35.6 (19.4),22.8 (16.3),13.2 (10.7),5.2 (4.3)
**Maggio et al., 2011 [12]**	7	Helical Tomotherapy	mpMRI (DWI+T2w+ T1w)	CTV = prostate + SV	PTV = CTV + 8-8-10 (in lateral, A-P and cranial-caudal direction respectively)<br>PTVboost = GTV + 5	[GTV]: Vmean = 2.7 (std:1.2), Vmedian = 2.8 (range:0.8~ 4.3)	PTV = 71.4 Gy	PTVboost = 80 Gy [153.7]	28	NR	NR	NR	[Mean/%]: V40=39.9,V50=29.3,V60=18.6
								PTVboost = 90 Gy [183.3]					NR
								PTVboost = 100 Gy [215.2]			GTV-[Mean/Gy]: D95=98.8<br>GTV-[Median/Gy]: D95=100.6		
								PTVboost = 120 Gy [285.9]			NR		

Nutting et al., 2002 [13]	6	IMRT	Histopathological data	CTV = prostate	PTV = CTV + 10	[DIL]:Vmean = 4.3 (std:2.1), Vmedian = 2.8 (range:1.9~ 7.2)	PTV = 70 Gy	GTV = 90 Gy [164.7]	35	PTV-[mean(SD)/Gy]: Dmean = 76.1 (1.8), Dmin = 67.4 (0.2), Dmax = 89.6 (1.2)	DIL-[mean(SD)/Gy]: Dmean = 89.1 (1.4), Dmin = 86.8 (1.4), Dmax = 92.7 (1.9)	[mean/Gy]: Dmax = 74.1 (1.9) [mean/%]: V90,80,50,20 = 21.1, 25.6, 42.5, 61.8	[mean/Gy]: Dmax = 81.8 (3.8) [mean/%]: V90,80,50,20 = 27.2, 36.6, 69.5, 90.6
						[non-DIL]:Vmean = 0.6 (std:0.3), Vmedian = 0.5 (range:0.2~ 1.2)				PTV-[mean(SD)/Gy]: Dmean = 74.7 (1.9), Dmin = 67.6 (0.3), Dmax = 88.6 (0.6)	DIL-[mean(SD)/Gy]: Dmean = 89.0 (0.2), Dmin = 86.3 (0.7), Dmax = 92.0 (1.1)	[mean/Gy]: Dmax = 74.9 (1.1) [mean/%]: V90,80,50,20 = 19.9, 24.3, 40.3, 59.6	[mean/Gy]: Dmax = 81.8 (1.6) [mean/%]: V90,80,50,20 = 28.7, 39.0, 69.7, 89.7
van Lin et al., 2006 [14]	5	IMRT	mpMRI (DCE+T2w) + MRSI(1H-spectroscopic MRI)	CTV = prostate	PTV =CTV + 7 PTVboost = GTV+5	Vmean = 2.7 cc (sd: 1.9), Vmedian = 1.8 (range:1.1~ 6.5)	PTV = 70 Gy	PTVboost = 90 Gy [164.7]	35	[mean(SD,range) /Gy]: Dmean = 71.5 (0.1, range: 71.3~71.6)	[mean]: Dmean = 90.6 (0.4, range: 90.0~91.2);	NR	NR
Zamboglou et al., 2018 [15]	10	IMRT	PET-CT (68Ga-PSMA)	CTV1 = Prostate + SV CTV2 = Prostate + partial SV GTVPET = PET-CT defined DILs	PTV1, PTV2, PTVPET, PTVMRI, and PTVunion were expanded by adding an isotropic margin of 4 mm to each CTV/GTV. GTVhist repesent the histology data defined GTV (i.e. IPLs). Volume of GTVhist: Vmean = 7.0 (std:6.4), Vmedian = 4.1 (range:1.4~19.8)	Vmean = 7.3 (std:4.4), Vmedian = 6.4 (range:1.9~ 15.5)	PTV2 = 77 Gy	GTVPET = 95 Gy [178.2]	35	NR	GTVhist-[Mean(SD)/Gy]: Dmean = 95.3 (2.6)	NR	NR
			mpMRI (T2w+DWI+DCE)	CTV1 = Prostate + SV CTV2 = Prostate + partial SV GTVMRI = mpMRI		Vmean = 4.8 (std:4.3), Vmedian = 2.6 (range:0.7~ 15.5)		GTVMRI = 95 Gy [178.2]			GTVhist-[Mean(SD)/Gy]: Dmean =93.3 (2.6)		

				defined DILs									
			PET-CT (68Ga-PSMA) + mpMRI (T2w+DWI+DCE)	CTV1 = Prostate + SV CTV2 = Prostate + partial SV GTVunion = GTVMRI + GTVPET		Vmean = 9.2 (std:5.6), Vmedian = 8.7 (range:2.2~22.2)		GTVunion = 95 Gy [178.2]			GTVhist-[Mean(SD)/Gy]: Dmean = 96.3 (1.5)		
			PET-CT (68Ga-PSMA)	CTV1 = Prostate + SV CTV2 = Prostate + partial SV GTVPET = PET-CT defined DILs		Vmean = 7.3 (std:4.4), Vmedian = 6.4 (range:1.9~15.5)		GTVPET = 80 Gy [134.3]			GTVhist-[Mean(SD)/Gy]: Dmean = :80.7 (0.4)		
			mpMRI (T2w+DWI+DCE)	CTV1 = Prostate + SV CTV2 = Prostate + partial SV GTVMRI = mpMRI defined DILs		Vmean = 4.8 (std:4.3), Vmedian = 2.6 (range:0.7~15.5)	PTV2 = 76 Gy	GTVMRI = 80 Gy [134.3]	38		GTVhist-[Mean(SD)/Gy]: Dmean = 79.9 (0.8)		
			PET-CT (68Ga-PSMA) + mpMRI (T2w+DWI+DCE)	CTV1 = Prostate + SV CTV2 = Prostate + partial SV GTVunion = GTVMRI + GTVPET		Vmean = 9.2 (std:5.6), Vmedian = 8.7 (range:2.2~22.2)		GTVunion = 80 Gy [134.3]			GTVhist-[Mean(SD)/Gy]: Dmean = 80.8 (0.5)		

**Blake et al., 2020 [16]**	12	IMRT	mpMRI (DWI+T2w+ DCE)	CTV1 = Prostate + SV<br>CTV2 = Prostate + involved SV<br>CTVboost = GTV + 3	PTV1 = CTV1 + 10<br>PTV2 = CTV2 + 5<br>PTVboost = CTVboost + 3	[PTVboost]: Vmean = 6.9 (std:5.0), Vmedian = 5.9 (range:2.2~20.1).	PTV2 = 74 Gy	PTVboost = 86 Gy [150.5]	37	NR	NR	[Median/Gy]: Dmean = 29.6, D1cc = 75.1	[Median/Gy]: Dmean = 36.8 Gy, D1cc = 74.6
**Pinkawa et al.,2010 [17]**	65	IMRT	PET-CT (18F-choline)	CTV = prostate	PTV = CTV + 8 (in lateral and anterior) + 5 (in cranial-caudal) + 4(posteriorly)<br>PTVboost = GTV + 4 (3 posteriorly)	[GTV] Vmean = 6.2	PTV = 76 Gy	PTVboost = 80 Gy [134.3]	38			NR	NR
**Pinkawa et al., 2009 [18]**	12	IMRT	PET-CT (18F-choline)	CTV = prostate volume (including base of seminal vesicles for intermediate and high risk patients)	PTV = CTV + 8 (in lateral and anterior) + 5 (in cranial-caudal) + 4(posteriorly)<br>PTVboost = GTV + 4 (3 posteriorly)	Vmean = 6 (range:1~16)	PTV = 76 Gy	PTVboost = 80 Gy [134.3]	38	PTV-[Mean(SD)/Gy]: Dmax = 83.2 (0.6); Dmean = 77.9 (0.8); Dmin = 73.0 (1.0)	PTVboost-[Mean(SD)/Gy]: Dmax = 81.9 (0.8); Dmean = 80.3 (0.3); Dmin = 78.4 (0.8)	[Mean(SD)/Gy]: Dmax = 78.4 (1.6); Dmean = 28.4 (10.3); Dmin = 2.1 (2.4)<br>[Mean(SD)/%]: V70 = 10.1 (6.1); V50 = 24.3 (12.4)	[Mean(SD)/Gy]: Dmax = 75.1 (2.9); Dmean = 34.5 (5.4); Dmin = 4.1 (4.6)<br>[Mean(SD)/%]: V70 = 6.4 (2.6); V50 = 24.0 (5.9)
							PTV = 66.6 Gy	PTVboost = 83.25 Gy [143.7]	37	PTV-[Mean(SD)/Gy]: Dmax = 85.7 (1.0); Dmean = 73.8 (3.4); Dmin = 65.8 (0.9)	PTVboost-[Mean(SD)/Gy]: Dmax =85.7 (1.0); Dmean = 83.3 (0.0); Dmin = 80.4 (1.2)	[Mean(SD)/Gy]: Dmax =72.8 (4.8); Dmean = 46.5 (7.1); Dmin = 0.7 (0.3)<br>[Mean(SD)/%]: V70 = 2.5 (2.8); V50 = 13.4 (9.9)	[Mean(SD)/Gy]: Dmax = 66.8 (7.1); Dmean = 25.5 (4.4); Dmin = 1.2 (0.5)<br>[Mean(SD)/%]: V70 = 1.5 (1.3); V50 = 13.8 (5.1)
**Ost et al., 2011 [19]**	12	IMRT (3 fields)	MRI + MRSI	CTV = prostate + SV	PTV = CTV + 4	NR	CTV = 72 Gy<br>PTV = 68 Gy	GTV = 80 Gy [134.3]	38	CTV-[Mean(SD)/Gy]: D98 = 75 (2); Dmean = 80 (2); Dmin = 86 (2)<br>PTV-[Mean(SD)/Gy]: D98 = 71 (2); Dmean = 78 (2); Dmin = 86 (2)	GTV-[Mean(SD)/Gy]: D98 = 82 (2); Dmean = 85 (2); Dmin = 86 (2);	[Mean(SD)/%]: V20 = 81 (11); V40 = 64 (13); V60 = 38 (10); V70 = 19 (6)	[Mean(SD)/%]: V40 = 41 (18); V70 = 8 (4)

		IMRT (5 fields)								CTV-[Mean(SD)/Gy]: D98 = 79 (2); Dmean = 86 (4); Dmin = 93 (5) PTV-[Mean(SD)/Gy]: D98 = 73 (2); Dmean = 84 (5); Dmin = 92 (6)	GTV-[Mean(SD)/Gy]: D98 = 89 (6); Dmean = 93 (6); Dmin = 94 (5);	[Mean(SD)/%]: V20 = 83 (11); V40 = 70 (11); V60 = 35 (7); V70 = 14 (5)	[Mean(SD)/%]: V40 = 40 (17); V70 = 9 (4)
		IMRT (7 fields)								[CTV/Mean(SD)]: D98 = 80 (3); Dmean = 88 (3); Dmin = 95 (5) PTV-[Mean(SD)/Gy]: D98 = 73 (2); Dmean = 85 (3); Dmin = 94 (5)	GTV-[Mean(SD)/Gy]: D98 = 89 (6); Dmean = 94 (5); Dmin = 96 (5);	[Mean(SD)/%]: V20 = 81 (11); V40 = 63 (12); V60 = 28 (7); V70 = 11 (5)	[Mean(SD)/%]: V40 = 34 (17); V70 = 8 (5)
		VMAT								CTV-[Mean(SD)/Gy]: D98 = 78 (3); Dmean = 88 (3); Dmin = 95 (5) PTV-[Mean(SD)/Gy]: D98 = 72 (2); Dmean = 85 (3); Dmin = 94 (5)	GTV-[Mean(SD)/Gy]: D98 = 89 (6); Dmean = 95 (5); Dmin = 96 (5);	[Mean(SD)/%]: V20 = 67 (8); V40 = 43 (8); V60 = 22 (5); V70 = 9 (2)	[Mean(SD)/%]: V40 = 30 (15); V70 = 8 (3)
**Bossart et al.,2016 [20]**	20	VMAT	mpMRI (T2w+T1w+DWI+DCE) or/and biopsy	CTV = prostate + proximal SV	PTV = CTV + 3-5	[GTV] Vmean = 3.9 (range:0.6~11.7)	PTV = 76 Gy	GTV = 89.3 Gy [157.0]	38	prostate-[Mean(range)/Gy]: D95 = 78.2 (76.5-81.1); Dmean = 84.4 (73.7-94.8)	GTV-[Mean (range)/Gy]: D95 = 89.7 (89.3-90.3)); Dmean = 91.7 (87.0-94.8)	[Mean (range)/%]: V40 = 33.3 (10.4-51.5); V65 = 13.9 (4.8-33.0); V80 = 3.7 (1.0-12.3)	[Mean (range)/%]: V40 = 26.8 (20.4-36.0); V65 = 9.6 (4.6-12.3); V80 = 0.8 (0.1-2.4)
	15					[GTV] Vmean = 2.5 (range:0.4~9.2)				prostate-[Mean(range)/Gy]: D95 = 77.8 (76.7-79.4); Dmean = 80.6 (76.0-94.7)	GTV-[Mean (range)/Gy]: D95 = 89.0 (88.9-89.4); Dmean = 91.5 (86.8-94.7)	[Mean (range)/%]: V40 = 16.9 (3.9-26.4); V65 = 6.7 (1.4-11.2); V80 = 1.3 (0.0-4.5)	[Mean (range)/%]: V40 = 16.3 (6.7-28.3); V65 = 4.0 (1.0-8.8); V80 =0.1 (0.0-0.2)
	5				PTV = CTV + 3-5 PTVboost = GTV + 3	NR		PTVboost = 89.3 Gy [157.0]		prostate-[Mean(range)/Gy]:D95 = 80.3 (77.9-81.7); Dmean = 85.0 (83.9-86.7)	GTV-[Mean (range)/Gy]: D95 = 93.2 (92.6-94.4); Dmean = 94.6 (93.9-96.9)	[Mean (range)/%]: V40 = 25.0 (20.2-31.9); V65 = 9.4 (8.1-10.8); V80 = 2.5 (1.6-3.7)	[Mean (range)/%]: V40 = 18.9 (14.5-24.4); V65 = 3.8 (2.5-5.7); V80 = 0.3 (0.0-0.7)

**De Meerleer et al., 2005 [21]**	15	IMRT	mpMRI (T1w+T2w)	CTV = prostate + SV	PTV = CTV + 7 (10 in cranial-caudal)	[GTV] Vmean = 10.5; Vmedian = 4 (range: 1-95)	CTV = 78 Gy PTV = 74 Gy	GTV = 80 Gy	NR	NR	NR	NR	NR
**Chang et al., 2012 [22]**	8	IMRT	PET-CT (11C-choline)	GTV60 = 60% of the maximum SUV GTV70 = 70% of the maximum SUV CTV1 = prostate + SV CTV2 = prostate	PTV1 = CTV1 + 6 PTV2 = CTV2 + 6 PTV60 = GTV60 + 6 PTV70 = GTV70 + 6	NR	PTV1 = 60 Gy PTV2 = 78 Gy	PTV60 = 80 Gy [132.9] PTV70 = 90 Gy	39	NR	NR	NR	NR
							PTV1 = 60 Gy PTV2 = 72 Gy	PTV60 = 84 Gy [142.4] PTV70 = 90 Gy					
**Kuang et al., 2015 [23]**	30	VMAT	PET-CT (18F-choline)	GTV60 = 60% of the maximum SUV GTV70 = 70% of the maximum SUV CTV = prostate + 1 cm of bilateral SV	PTV = CTV+ 6 (3 in cranial-caudal) PTV60/PTV70 = GTV60/GTV70 + 6 (3 in cranial-caudal)	[GTV60]: Vmedian = 2.7 (range: 0.3-10.8); [GTV70]: Vmedian = 0.7 (range: 0.04-7.10)	PTV =76 Gy	PTV60 = 100 Gy [182.7] PTV70 = 105 Gy	39	PTV-[Median(range)/Gy]: D95 =79.5 (79.0~80.2); Dmean = 90.0 (84.5~92.4); Dmax = 108.3 (106.6~110.8)	PTV60-[Median(range)/Gy]: D95 =102.3 (100.3~104.8); Dmean = 105.8 (102.5~107.0); Dmax = 109.6 (107.5~114.6); Dmin = 97.6 (86.5~99.4)	[Median(range)/Gy]: Dmax (range) = 83.2 (81.2~86.7); Dmean = 34.3 (8.4~51.2) [Median(range)/%]: V80,75,70,65 = 1.6,7.0,9.2,12.1	[Median(range)/Gy]: Dmax = 83.6 (64.5~89.0); Dmean = 35.6 (24.6~46.6) [Median(range)/%]: V75,70,65,60,50 = 1.6,3.4,6.3,10.9,24.2
**Seppälä, Seppänen, Arponen, Lindholm, & Minn, 2009 [24]**	12	IMRT	PET-CT (Carbon-11)	CTV = prostate	PTV = CTV + 6 PTVboost = GTV + 6	[GTV] Vmean = 5.4 (std:4.5), Vmedian = 3.1 (range:0.8~13.9)	PTV = 72.2 Gy	PTVboost = 77.9 Gy, 81 Gy, 84 Gy, 87 Gy and 90 Gy	41	NR	NR	NR	NR
**Housri et al., 2011 [25]**	24	IMRT	mpMRI (T1w+T2w+DWI+DCE+MRS) + MRSI	CTV = prostate	PTVboost = GTV + 3 PTV = prostate + 9 (5 posteriorly)	[GTV]: Vmean = 1.57	PTV = 75.6 Gy	PTVboost = 151.2 Gy (200% of the prescribed dose)	42	NR	NR	NR	NR

**Abedi, Tavakkoli, Jabbari, Amouheidari, & Yadegarfard, 2017 [26]**	24	IMRT	mpMRI (DWI+DCE) + MRSI + histopathological data	CTV1 = Prostate + SV<br>CTV2 = Prostate	PTV1 = CTV1 + 5<br>PTV2 = CTV2 + 5<br>PTVboost = GTV+ 5	NR	PTV2 = 78 Gy	PTVboost = 90 Gy [148.1]	45	NR	NR	[Mean/Gy]: D80 = 28, D65 = 21	[Mean/Gy]: D75 = 17, D50 = 22
**Yeo et al.,2015 [27]**	10	IMPT	hypothetical IPLs	PTV1 = SV + 5<br>PTV2 = prostate + 5<br>PTVboost = GTV + 5		[GTV] Vmean = 4cc	PTV1 = 50.4 Gy<br>PTV2 = 70.2 Gy	PTVboost = 90 Gy [142.3]	50	PTV2-[Mean/Gy]: Dmin = 69.1; Dmax = 93.4; Dmean = 59.9	PTVboost-[Mean/Gy]: Dmin = 89.4; Dmax = 93.6; Dmean = 82.4	[Mean/Gy]: Dmean = 19.7<br>[Mean/%]: V90,80,75,70,65 = 2.8, 8.1, 10.0, 12.1, 14.1	[Mean/Gy]: Dmean =21.5<br>[Mean/%]: V90,75,70,65,60 = 1.1, 6.7, 9.7, 12.3, 14.6
		IMRT								PTV2-[Mean/Gy]: Dmin = 67.5; Dmax = 94.77; Dmean = 57.4	PTVboost-[Mean/Gy]:Dmin = 88.0; Dmax = 97.1; Dmean = 84.5	[Mean/Gy]: Dmean = 24.5<br>[Mean/%]: V90,80,75,70,65 = 1.7, 5.0, 6.7, 8.6, 10.5	[Mean/Gy]: Dmean =38.9<br>[Mean/%]: V90,75,70,65,60 = 0.7, 7.3, 11.4, 15.7, 20.4
**Kim et al., 2008 [28]**	15	Brachytherapy	MRI (T2w) + MRSI	PTV included the prostate only for T1c-T2b and the prostate and extra-capsular extension for T3a-T3b.		[GTV] Vmean (std) = 6.3 cc (4.3 cc); Vmin = 1.2 cc; Vmax = 15.3 cc	100% prescribed dose = 9.5 Gy	GTV = 10.45~14.25 Gy;	2	PTV-[Mean(SD,range)/%]: V100= 93.7 (1.1, 91.9~94.9); V150 = 40.4 (3.9, 31.8~45.5)	[Mean(SD,range)/%]: V120 = 99.0 (2.5, 91.4~100.0), V150 = 82.4 (21.2, 39.2~99.2), V200 = 33.2 (16.2, 11.1~59.4)	[Mean(SD,range)/cc]: V75% = 0.53 (0.36, 0.02~0.98)	[Mean(SD,range)/cc]: V75% = 0.63 (0.24, 0.16~0.99)
**Pouliot et al., 2004 [29]**	10	Brachytherapy	MRI (T1w+T2w) and MRSI	The treatment planning includes generating dose distributions for 3 different boost levels (B1, B2, B3)		Vmean = 3.6 (std:1.2), Vmedian = 3.3 (range:2.0~5.8)	Dmin and Dmax for prostate = 100% and 150% of prescribed dose	Dmin and Dmax for GTV = 120% and 150% of prescribed dose;		Prostate-[Mean/%]: V150% = 40.4	GTV-[Mean/%]: V120% = 97.1	[Mean/cc]: V50% = 3.4cc; V80% = 0.6cc	[Mean/cc]: V50% = 5.8; V80% = 0.8
								Dmin and Dmax for GTV = 150% and 150% of prescribed dose;		Prostate-[Mean/%]: V150% = 49.3	GTV-[Mean/%]: V150% = 77.8	[Mean/cc]: V50% = 3.5; V80% = 0.6	[Mean/cc]: V50% = 6.5; V80% = 1.2
								Dmin and Dmax for GTV = 150% and 170% of prescribed dose;		Prostate-[Mean/%]: V150% = 50.6	GTV-[Mean/%]: V150% = 86.1	[Mean/cc]: V50% = 3.5; V80% = 0.6	[Mean/cc]: V50% = 6.6; V80% = 1.2

**Crook et al., 2014 [30]**	22	Brachytherapy	mpMRI (T2w+DCE)	CTV (for intermediate risk) = prostate + SV<br>CTV (for high risk) = prostate + SV + pelvic lymph nodes	PTV = CTV + 10 (7 posteriorly)	Vmean = 2.9 (SD: 1.8)<br>Vmedian = 7.6 (range: 2.7~39.8)	100% prescribed dose = 10 Gy	GTV = 12.5 Gy	2	[mean(SD)/Gy]: D90 = 10.9 (0.2)	[mean(SD)/Gy]: D90 = 13.2 (1.1)<br>[mean(range)/Gy]: D90 = 13.1 (11.7~17.9)	NR	Dose to 1 cc of rectal wall = 6.4Gy
**Kazi, Godwin, Simpson, & Sasso,2010 [31]**	1	EBRT+HDR Brachytherapy	MRI (T2w) and MRSI	CTV1 (delineated on CT) = prostate + base of SV<br>CTV2 = prostate (delineated on MRI)	PTV1 = CTV1 + 5(P) + 10 (all rest directions), PTV1 not exclude DIL.	NR	PTV1 = 60 Gy/30fx (by EBRT)<br>CTV2 = 7.5 Gy (By HDR)	GTV = 15 Gy (by HDR)	1	For HDR brachytherapy and for CTV2: Dmean (Dmin,Dmax) = 14.9 (7.1-241.0) Gy; D90,100 = 9.0, 7.5 Gy	For HDR brachytherapy and for GTV: Dmean (Dmin,Dmax) = 29.8 (15.1-124.7) Gy; D90,100 = 18.4,15.8Gy	NR	NR
**DiBiase et al., 2002 [32]**	14	Brachytherapy	MRSI + T2w	CTV = prostate	PTV = CTV + 2–3 mm margin in all dimensions except posteriorly	NR	100% prescribed dose= 145 Gy	GTV = 188 Gy, 130% of the prescribed dose		NR	NR	NR	[Mean(range)/%]: Dmax = 110 (74–150)
**Mason et al.,2014 [33]**	15	Brachytherapy	mpMRI (DWI+T2w+DCE)	CTV = prostate	PTV1 = CTV + 3 (0 posteriorly)<br>PTVboost = GTV + 4.5	[GTV] Vmedian = 1.9 cc (range, 0.4~23.0 cc)	100% prescribed dose = 15 Gy	aims to achieved 150~200% of prescribed dose to PTVboost	1	CTV-[Median(range)/%]: D90%= 16.9 (16.6~17.6); V100% = 99.5 (98.5~99.8); V150% = 34.8 (24.3~44.9); V200% = 8.8 (6.2~13.6)	GTV-[Median(range)/Gy]: D90 = 22.6 (17.5~32.3)<br>-[Median(range)/%]: V100% = 100; V150% = 90.9 (32.2~100); V200% = 26.4 (10.0~100)	NR	[Median(range)/Gy]: D2cc = 9.0 (6.8~10.4)
		Brachytherapy (with additional HDR needles)								CTV-[Median(range)/%]: D90% = 17.0 (16.2~17.5); V100% = 99.4 (98.0~99.9); V150% = 40.7 (24.6~52.3); V200% = 10.8 (5.3~16.7)	GTV-[Median(range)/Gy]: D90 = 23.4 (17.9~37.5)<br>-[Median(range)/%]: V100% = 100; V150% = 99.2 (39.2~100); V200% = 40 (2.7~99.5)		[Median(range)/Gy]: D2cc = 9.0 (6.6~10.2)

Dankulchai et al.,2014 [34]	13	Brachytherapy	mpMRI (DWI+T2w+DCE) + MRSI	CTV = prostate	PTV1 = CTV + 3<br>PTVboost = GTV + 3<br>PTV2 = PTV1 - PTVboost	[GTV] Vmedian = 1.6 cc (range:0.1~6.1 cc)	PTV2 = 19 Gy	PTVboost = 21 Gy	1	NR	NR	NR	NR

Table S2

Table S2: Summarized characteristics of reviewed trials.

**Author, year**	**N**	**Risk group**			**Hormone therapy**	**Initial Median PSA (mg/mL)**	**Lesion volume (cc)**	**GTV Identification modality**	**Treatment Modality**	**Volume delineation and margins (mm)**	**Prescription Dose and Fractions (fx)**	**Median Follow-up time**	**Acute G2+ toxicity (G3)**		**Late G2+ toxicity**		**Clinical Outcome (or dosimetric outcome)**
		L	I	H									**GU**	**GI**	**GU**	**GI**	
Tree et al., 2023 [35]	55	0%	73%	27%	100 %	11 (IQR: 7.4-17)	NR	mpMRI (T2w + DWI)	IMRT + VMAT	PTV1 = (prostate + SV) + 6<br>PTV2 = (prostate + based of SV) + 6 (3 posteriorly)<br>PTV3 = (prostate + based of SV) + 3 (0 posteriorly)<br>PTVboost = GTV + 2<br>PTV4 = pelvic lymph node + 5	PTV1 = 60/37 fx<br>PTV2 = 71/37 fx<br>PTV3 =74 Gy/37 fx<br>GTV = 82 Gy/37 fx	5 years	38.20%	10.90%	16.6% (3.7%)	12.8% (0%)	5-year bDFS = 98.2%
	153	0%	46%	54%		10.7 (IQR: 7.6-15.0)					PTV1 = 48.6/20 fx<br>PTV2 = 57.6/20 fx<br>Prostate =60 Gy/20 fx<br>GTV = 67 Gy/20 fx		38.90%	13.70%	20.2% (2%)	15.5% (0.9%)	5-year bDFS = 96.7%
	48	0%	0%	100 %		15.5 (IQR: 7.9-26.7)					PTV4 = 60 Gy/37fx<br>PTV1 = 60/37 fx<br>PTV2 = 71/37 fx<br>PTV3 =74 Gy/37 fx<br>GTV = 82 Gy/37 fx		37.50%	14.60%	20.7% (2.5%)	23.4% (2.7%)	5-year bDFS = 95.1%

**Eade et al., 2022 [36]**	112	2%	79%	20%	5%	NR	NR	PET-CT + mpMRI	IMRT	PTV = CTV (prostate + proximal SV) + 5 (3 posteriorly)	GTV = 45 Gy/5fx<br>CTV = 40 Gy/5fx<br>PTV = 36.25 Gy/5fx	2.3 years	NR	NR	NR	NR	3-year estimated BF for IR= 1%<br>3-year estimated BF for HR= 15%
**Strnad et al., 2022 [37]**	101	22%	31%	48%	32%	NR	NR	TRUS	IMRT + PDR- OR HDR-Brachytherapy	[EBRT]: PTV = (prostate + SV) + 5-10	[EBRT]: PTV = up to 50.4 Gy/28fx<br>[PDR]: prsotate = 35 Gy in 1 session; GTV = 40 Gy/2fx<br>[HDR]: prostate = 9-9.5 Gy/2fx; GTV = 21.6-22.8 Gy/2fx	[Median]: 65 months	NR	NR	9% (4%)	0%	The cumulative 5 years local recurrence rate (LRR) for all patients was 1%
									PDR- OR HDR-Brachytherapy		[PDR]: prsotate = 70 Gy in 2 sessions; GTV = 84 Gy in 2 sessions<br>[HDR]: prostate = 36 Gy in 2 sessions; GTV = 43.2 Gy in 2 sessions						
**Dankulchai et al., 2022 [38]**	45	7%	36%	58%	100 %	12.3 (IQR:8.6–23.7)	NR	mpMRI (T2w + DWI + DCE)	VMAT	PTV = (prostate + SV + GTV) + 5 (3 posteriorly)	GTV = 87.75 Gy/39fx OR 70 Gy/20fx<br>prostate = 78 Gy/39fx OR 60 Gy/20fx	20 (IQR: 10–25) months	28.9% (2.2%)	8.9% (2.2%)	8.9% (0%)	2.2% (0%)	
**Zamboglou et al., 2022 [39]**	25	0%	24%	76%	32%	7.5 (range: 3.9 - 24.9)	Vmedian = 3.8 (IQR: 3.0-6.6)	PET-CT and mpMRI (T2w + DWI + DCE)	IMRT	PTV1 = CTV1 (prostate + base of SV) + 6<br>PTV2 = CTV2 (prostate) + 4<br>PTVboost = GTV + 2-4	PTV1 = 45 Gy/14 fx<br>PTV2 = 18 Gy/6 fx<br>PTVboost = up to 75 Gy or 63 Gy/20 fx	6 months	NR	NR	36% (0%)	16% (0%)	PTVboost- [Median(range)/Gy]: Dmean = 70 (range: 64-75)

	25	0%	36%	64%	44%	7.4 (2.6 - 26.9)			IMRT + HDR Brachyt herapy	PTV1 = CTV1 (prostate + base of SV) PTV2 = CTV2 (prostate (for ≥ cT2c: + base of SV)) PTVboost = GTV + 2	[HDR]: PTV1 = 15 Gy/1fx PTVboost = 17.5-19 Gy/1fx		NR	NR	48% (0%)	12% (0%)	PTVboost-[Median(range)/Gy]: D90 = 19 (range: 15-21)
**Hannan et al., 2022 [40]**	12	0%	0%	100 %	100 %	9.7 (IQR, 5.8-19.9)	NR	mpMRI (T2w)	VMAT	PTV1 = pelvic lymph node + 5 PTV2 = (prostate+proximal SV) + 3 PTVboost = GTV + 0-3	PTV1 = 22.5 Gy/5fx PTV2 = 47.5 Gy/5fx PTVboost = 50 Gy/5fx	42 (range: 30-48) months	25% (0%)	13% (0%)	22% (2%)	7% (0%)	PTVboost-[Mean/Gy]: D99 = 50; PTVboost-[Median(IQR)/Gy]: D99 = 49 (50-50)
	15										PTV1 = 25 Gy/5fx PTV2 = 47.5 Gy/5fx PTVboost = 50 Gy/5fx	24 (range: 12-36) months					PTVboost-[Mean/Gy]: D99 = 50; PTVboost-[Median(IQR)/Gy]: D99 = 50 (49-50)
	13										PTV1 = 25Gy/5fx PTV2 = 47.5 Gy/5fx PTVboost = 52.5 Gy/5fx	12 (range: 12-18) months					PTVboost-[Mean/Gy]: D99 = 51; PTVboost-[Median(IQR)/Gy]: D99 = 51 (52-52)
	15										PTV1 = 25 Gy/5fx PTV2 = 47.5 Gy/5fx PTVboost = 55 Gy/5fx	7.5 (range: 3-9) months					PTVboost-[Mean/Gy]: D99 = 51; PTVboost-[Median(IQR)/Gy]: D99 = 49 (51-54)
**Kerkmeijer et al., 2021 [41]**	287	1%	15%	84%	65%	15.2 (SD: 14.9)	NR	NA	IMRT	PTV = CTV (prostate + CV) + 5-8	PTV = 77 Gy/35fx	72 (IQR: 58-86) months	46.00%	10.10%	23.0% (3.5%)	12.2% (1.4%)	5-year bDFS: 85% (38 events; 95% CI, 80 to 89)
	284	1%	15%	84%	65%	16.3 (SD: 13.9)	NR	mpMRI (T2w + DWI + DCE)	IMRT, VMAT		PTV = 77 Gy/35fx GTV = 95 Gy/35fx		42.30%	14.80%	27.8% (5.6%)	12.7% (1.4%)	5-year bDFS: 92%(21 events; 95% CI, 87 to 94)

Zapatero et al., 2021 [42]	30	13%	50%	37%	50%	8.5 (IQR: 5.5–14.5)	NR	mpMRI (T2w + DWI + DCE)	VMAT	PTV = CTV (prostate + proximal SV) + 7-9 (5-7 posteriorly) PTVboost = GTV + 3 (2 posteriorly)	PTV = 76 Gy/35fx PTVboost = 85.05 Gy/35fx	30.0 (IQR: 25.5–40.27) months	20% (0%)	0%	0%	0%	biochemical relapse = 0% PTV- [Median(IQR)/Gy]: Dmedian = 77.6 (77.3–78.1) PTVboost- [Median(IQR)/Gy]: Dmedian = 85.2 (85.0–85.4) GTV- [Median(IQR)/Gy]: Dmedian = 85.5 (85.0–86.0)
Rezaeijo et al., 2021 [43]	20	0%	45%	55%	100 %	Mean PSA: 24.86 (SD: 9.5)	Vmean = 1.73 (SD: 1.41)	mpMRI (T2w + DWI + DCE)	IMRT	PTV = prstate + 7 (6 posteriorly) PTVboost = GTV + 5	PTV = 80 Gy/40fx PTVboost = 80, 85, 91Gy/40fx	NR	NR	NR	NR	NR	Average DIL shrinkage: 98.83% (range: 78.9%–100%)
	20	0%	40%	62%	100 %	Mean PSA: 22.22 (SD: 10.8)	Vmean = 1.67 (SD: 1.45)	NA		PTV = prstate + 7 (6 posteriorly)	PTV = 80 Gy/40fx						Average DIL shrinkage: 81.95% (range: 38.89%–100%)
Kuisma et al., 2021 [44]	19 (Total:30)	17%	57%	27%	0%	NR	Vmedian = 2.6 (range: 0.3–13.0)	PET-CT (11C)	EBRT	PTV = prostate + 8-10 PTVboost = GTV + 0-6	PTV =72.9 Gy/38fx PTVboost = 79.4 Gy/38fx Prostate = 76.6 Gy/38 fx GTV = 80.4 Gy/38 fx	124 (range: 105–137) months	6.7% (0%)	16.7% (0%)	17.4% (0%) N=23	4.2% (4.2%) N=24	PTVboost- [Median(range)/Gy]: Dmedian = 79.3 (78.0–80.1) GTV- [Median(range)/Gy]: Dmedian = 80.2 (79.1–81.2)

	11 (Total:30)						Vmedian = 8.0 (range: 0.5–23.3)										PTVboost- [Median(range)/Gy]: Dmedian = 79.5 (78.0–80.2)<br>GTV- [Median(range)/Gy]: Dmedian = 80.7 (80.1–81.7)
**Armstrong et al., 2021 [45]**	25	0%	44%	56%	100 %	NR	Vmean = 3.9 (range: 1.2–9.6)	mpMRI (T2w + DWI + DCE)	HDR Brachytherapy	PTV = prostate + 3<br>PTVboost = GTV + 2	GTV = 21 Gy/1 fx<br>PTV = V15Gy>95%; 65%<V19Gy<75 %	75 (range: 66–85) months	16%(0 %)	0%	RiF(6%)	RiF	PTV- [Mean(range)/Gy]: D90 = 16.73 (15.88–17.39); V19 = 72.9 (65.2–74.9)<br>PTVboost- [Mean(range)/Gy]: D90 = 23.49 (21.79–25.78); Dmean = 30.3 (28.0–38.5)<br>BF = 32%; 5-year bNED = 88%
	25	4%	40%	56%	96%	NR	Vmean = 3.3 (range: 1.2–12.1)				GTV = 21 Gy<br>PTV = V15Gy>95%; V19Gy<50%	57 (range: 20–71) months					PTV- [Mean(range)/Gy]: D90 = 16.09 (15.63–16.87); V19 = 58.3 (45.7–78.8)<br>PTVboost- [Mean(range)/Gy]: D90 = 22.96 (21.78–24.82); Dmean = 30.2 (27.5–35.8)<br>BF = 28%; 5-year bNED = 76%
**Sanmamed et al., 2020 [46]**	40	20%	70%	10%	0%	8.3 (SD: 4)	Vmean = 2.2 (SD: 2.2)	mpMRI (T2w + DCE + DWI)	VMAT	PTV =prostate + 5 (3 laterally)	PTV = 76Gy/38fx<br>GTV = up to 95Gy/38fx	30 (range: 26–44) months	32.5% (2.5%)	10.0% (2.5%)	27.5% (2.5%)	7.5% (2.5%)	GTV- [Mean(SD)/Gy]: D99 = 105.3 Gy (2.8)

	40	12%	83%	5%	5%	8.1 (SD: 3)	Vmean = 3 (SD: 1.8)		VMAT + HDR brachytherapy	PTV =prostate + 1 (2 in cranial-caudal)	[VMAT]: PTV = 76Gy/38fx [HDR]: GTV = 10Gy/1fx	33 (range: 24–48) months	40.0% (2.5%)	10% (0.0%)	17.5% (0.0%)	7.5% (0.0%)	GTV- [Mean(SD)/Gy]: D99 = 103.1 Gy (6.7)
**Nicholls et al., 2020 [47]**	8	0%	NR	NR	100 %	7.4 ng/ml (range: 4.7–10.8)	Vmedian = 0.6 (range: 0.3–3.5)	mpMRI (T2w + DWI + DCE)	CyberKnife	PTV = CTV(prostate + proximal SV) + 5 (3 posteriorly)	PTV = 36.25/5 fx GTV = 40 Gy (max:47.5)/5fx	56 (range 50–74) months	37.50%	37.50%	12.50%	0%	PTV- [Median(range)/Gy]: D95 = 36.55 (35.87–36.99) GTV- [Median(range)/Gy]: D95 = 46.62 (44.85–48.25)
**Murray et al., 2020 [48]**	55	0%	73%	27%	100 %	11 (IQR: 7.4-17)	Vmedian = 2.4 (IQR: 1.3-4.0)	mpMRI	IMRT	PTV1 = (prostate + SV) + 6 PTV2 = (prostate + based of SV) + 6 (3 posteriorly) PTV3 = (prostate + based of SV) + 3 (0 posteriorly) PTVboost = GTV + 2	PTV1 = 60/37 fx PTV2 = 71/37 fx PTV3 =74 Gy/37 fx GTV = 82 Gy/37 fx	74.5 months	RiF	11%	9.1% (1.8%)	12.8% (0%)	Biological progression = 7% PTVboost - [Mean(SD)/Gy]: Dmean = 82.1 (0.12)
	50	0%	60%	40%	100 %	11.8 (IQR: 7.9-17)	Vmedian = 1.7 (IQR: 0.9-2.9)				PTV1 = 48.6/20 fx PTV2 = 57.6/20 fx Prostate =60 Gy/20 fx GTV = 67 Gy/20 fx	52 months	RiF	10%	22.1% (0%)	14.0% (0%)	PTVboost - [Mean(SD)/Gy]: Dmean = 67.0 (0.02)
**Marvaso et al., 2020 [49]**	64	20%	80%	0%	8%	6.07 (IQR: 4.79-7.98)	NR	mpMRI	EBRT	PTV = prostate + 5 (3 posteriorly) PTVboost = GTV + 3	PTV = 36.25 Gy/5 fx PTVboost = 37.5 Gy in 5 fx	NR	1.54% (0%)	3.08% (0%)	4.69% (0%)	3.13% (1.56%)	2 years b-PFS = 97% OS = 98% biochemical and clinical relapse = 2 patients
**Draulans et al., 2020 [50]**	100	0(0.0 %)	25%	75%	62%	10.8 (range: 3.0–29.0)	Vmedian = 2.3 (range: 0.1-27.8)	mpMRI (T2w + DWI + DCE)	VMAT	CTVboost = GTV+ 4mm PTV = CTV + 4 (n = 63) or 5 (n = 37)	PTV = 33.25 Gy/5fx GTV = 35 Gy with an iso-toxic boost up to 50 Gy/5fx	18 (6–30) months	34.0% (0.0%)	5.0% (0.0%)	14% (0%)	4% (0%)	GTV- [Median(range)/Gy]: Dmean = 44.7 (37.7–50.9); D99 =40.3 (36.2-50.7); Dmax = 48.2 (39.6-56.2)

Alayed et al., 2020 [51]	30	0%	0%	100 %	100 %	10.82 (range: 1.4-37.4)	NR	NA	EBRT	PTV1 = CTV1 (pelvic lymph nodes + SV) + 6 PTV2 = CTV2 (Prostate) + 3	CTV1 = 25Gy/5fx CTV2 = 40Gy/5fx PTV1 = 23.75Gy/5fx PTV2 = 40Gy/5fx	60 months	43.3% (0%)	3.3% (0%)	46.7% (0%)	23.3% (0%)	BF = 0%
	30	0%	37%	64%		11.97 range: (0.01-43.2)		mpMRI (T2w + DWI + DCE)		PTV1 = CTV1 (pelvic lymph nodes + SV) + 6 PTV2 = CTV2 (Prostate) + 2 (2.5 in cranial-caudal)	CTV1 = 25Gy/5fx CTV2 = 35Gy/5fx PTV1 = 23.75Gy/5fx PTV2 = 33.25Gy/5fx GTV = 50Gy/5fx	25 months	67% (3.3%)	16.7% (0%)	46.7% (0%)	13.3% (0%)	GTV-[Median(range)/Gy]: D90 = 48.3 (42.5-51.9); D99 = 44.6 (39.1-49.6)
Pollack et al., 2020 [51]	25	0%	0%	100 %	14 (56%)	5.8 (range: 3.1-18.9)	Vmean = 2.5 (range: 0.60-8.06)	mpMRI (T1w + T2w + DWI + DCE)	CyberKnife + IMRT	PTV = (Ptostate + SV) + 3-5	PTV = 76 Gy/38fx GTV = 88-90 Gy/38fx (12-14 Gy in day 1)	66.2 (range: 20.8-71.1) months	52% (0%)	20% (0%)	12% (G4: 4%)	16% (0%)	BF = 8%; bFFS: 92% GTV-[Mean(range)/Gy]: D95 = 7.62 (1.69-12.58) (day 1)
Herrera et al., 2019 [52]	9	35%	65%	0%	5%	12.3 (range, 2.7-40)	Vmedian = 2 (range: 0.6-4)	mpMRI (T2w)	CyberKnife	PTV = prostate + 3 PTVboost = GTV + 3	PTV = 36.25Gy/5fx PTVboost = 45, 47.5, or 50 Gy/5 fx	24 (range: 6-39) months	25% (0.0%)	10% (0.0%)	5.0% (0.0%)	0.0% (0.0%)	PTVboost-[Median(range)/Gy]: Dmean = 50 (47-55); D95 = 45 (40-50.4)
	11																PTVboost-[Median(range)/Gy]: Dmean = 53 (50-57); D95 = 48 (40-51.3)
McDonald et al., 2019 [53]	26	23%	77%	0%	31%	6.1 (range: 2.5-17.6)	Vmedian = 2.1 (range: 0.1-6.2)	MRI (T1, T2)	EBRT	PTV = prostat +5 (3 posteriorly) PTVboost = CTVboost (GTV + 5)+5(3 posteriorly)	PTV = 36.25Gy/5fx PTVboost = 40Gy/5fx	< 90 days	NR	NR	NR	NR	PTVboost-[Median(range)/%]: V40Gy = 88 (50.2-100); PTV-[Median(range)/%]: V36.25Gy = 95.1 (43.3-99.9);
Guimond et al., 2019 [54]	110	0%	100 %	0%	7%	6.3 (range:3.2–10.1)	NA	NA	LDR Brachytherapy (I-125 seeds)	PTV = prostate + 3	PTV = 144 Gy (100%)	82 (range: 60–100) months	33.3% (0%)	0%	36.6% (0%)	0%	Estimated 7-year bDFS: 89%

	55	0%	100 %	0%	22%	6.1 (4.1–11.0)	Vmedian = 4.3 (range: 2.6–6.2)	double-sextant biopsies + TRUS			PTV = 144 Gy (100%)<br>GTV = 216 Gy (150%)	71 (range: 60–92) months	46.2% (0%)	0%	39.7%(2%)	0%	Estimated 7-year bDFS: 96%
Alayed et al., 2019 [55]	60	8%	92%	0%	0%	6.24 (range: 2.2, 16.4)	Vmedian = 3.1	mpMRI (T2w + DWI + DCE)	HDR Brachytherapy		Prostate = 19 Gy/1 fx<br>GTV = 23 Gy/1 fx	39 months	21.7% (0%)	0% (0%)	15% (0%)	1.6% (0%)	BF = 18.3%;<br>GTV-[Median(range)/Gy]: D90 = 27.2 (20.9-35.7)<br>Prostate-[Median(range)/%]: V100% = 96.9% (93.8, 99.7)
Timon et al., 2018 [56]	13	23%	77%	0%	8%	5.8 (range: 4.3–17)	Vmedian = 1.18 (range: 0.20–5.40)	mpMRI (T2w + DWI + DCE)	EBRT	PTV = prostate + 5 (3 posteriorly)<br>PTVboost = GTV + 3	PTV = 36.25 Gy/5fx<br>PTVboost = 37.5 Gy/5fx	17 (range: 11–26) months	7.70%	0%	NR	NR	Median D95, D95, and D98 of the PTV, PTVboost, and GTV were: 97.7%, 99.2 and 101.7%,
Schlenter et al., 2018 [57]	67	42%	27%	31%	22%	6 (range: 2–53)	NA	NA	IMRT	PTV = CTV (prostate + based of SV) + 8 (5 in cranial-caudal, 4 posteriorly)	PTV = 76 Gy/38fx	64 (Mean: 58)	NR	NR	NR	NR	5-year biochemical tumor control = 85%<br>5-year OS = 88%
	67	43%	37%	19%	13%	7 (range: 2–83)	Vmedian = 4 (range: 0.4–37)	PET-CT		PTV = CTV (prostate + based of SV) + 8 (5 in cranial-caudal, 4 posteriorly)<br>PTVboost = GTV + 4	PTV = 76 Gy/38fx<br>PTVboost = 80 Gy/38fx	66 (Mean: 60)					5-year biochemical tumor control = 92%<br>5-year OS = 100%
Onjukka et al., 2017 [58]	28	0%	0%	100 %	100 %	12 (range: 4.6-59)	Vmedian = 4.3 (range: 0.46-15)	mpMRI (DWI)	VMAT	PTV1 = CTV1 (Prostate + SV) + 9<br>PTV2 = CTV2 (prostate + base of SV) + 5<br>PTVboost = CTVboost (GTV + 3) + 2	PTV1 = 53 Gy/20fx<br>PTV2 = 60 Gy/20fx<br>PTVboost = up limit 68 Gy	38 (range: 32 to 45) months	0%	0%	7.1% (0%)	0%	3 patients relapsed<br>PTV2-[Mean(range)/Gy]: Dmean = 61 (61-63); D98 = 59 (58-60)<br>PTVboost-[Mean(range)/Gy]: Dmedian = 67 (63-71); D98 = 63 (58-65)

**Uzan, Nahum, & Syndikus, 2016 [59]**	11	0%	0%	100 %	100 %	[Mean]: 15.9 (range: 6.8-51)	Vmean = 5.3 (range: 1.9-11.1)	functional MRI	IMRT (5 beams)	PTV1 = CTV1 (Prostate + SV) + 9 PTV2 = CTV2 (prostate + base of SV) + 5 (2 posteriorly) PTVboost = CTVboost (GTV + 3) + 2	PTV1 = 64 in 37fx PTV2 = 74 in 37fx PTVboost = Radiobiologically optimised	[Mean]: 36 (range: 24-50) months	NR (0%)	9.1% (0%)	18.2% (0%)	0%	PTV2-[Mean(range)/Gy]: Dmedian = 75.4 (75.1-75.8); PTVboost-[Mean(range)/Gy]: Dmedian = 86.4 (80.3-90.9); One patient relapsed
**Sundahl et al., 2016 [60]**	185	10%	50%	40%	100 %	NR	NA	NA	IMRT	PTV = CTV(prostate + SV) + 3	PTV = 78 Gy/38fx	72 (ramge: 6–144) months	NR	33%	NR	13%	
	225	2%	43%	55%	100 %		Vmean = 4.11 (range: 1–42)	mpMRI (T2w + DWI + DCE) + MRSI			PTV = 78 Gy/38fx GTV = 82 Gy/38fx	60 (ramge: 1–132) months		29%		10%	6-years bRFS = 84±3%
**King et al., 2016 [61]**	47	74%	26%	0%	17%	5.1 (IQR: 3.8-7.0)	Vmedian = 0.29	mpMRI (T2w + T1w) + MRSI	LDR brachytherapy (125I)		CTV = 144 Gy (100%) GTV = 216 Gy (150%)	86.4 (IQR: 49.8-117.6) months	60% (0%)	32% (11%)	4% (0%)	15% (2%)	10-year PSA relapseefree survival estimates were 100% and 89% (71-100%) for low- and intermediaterisk patients
**Gomez-Iturriaga et al., 2016 [62]**	15	0%	100 %	0%	NR	NR	Vmedian = 1.4 (range: 1.3–18)	mpMRI (T2w + T1w +DWI) + MRSI	EBRT + HDR brachytherapy		[EBRT]: prostate = 37.5 Gy/5fx [HDR]: prostate = 15 Gy/1fx GTV = 18.75 Gy/1fx	18 (range: 17–24) months	20% (0%)	13.4% (0%)	6.7% (0%)	0%	Prostate-[Median(range)/Gy]: D90 = 110.7 (107.9–113.6) GTV-[Median(range)/Gy]: D90 = 142.7 (131.4–151.7)
**Crook et al. 2014 [30]**	22	NR	NR	NR	NR	Mean PSA: 10.4 (SD: 4.4)	Vmean = 2.9 (SD: 1.8)	mpMRI (T2w + DWI + DCE)	EBRT + HDR brachytherapy		[EBRT]: prostate = 46 Gy/23fx [HDR]: prostate = 10 Gy/1fx GTV = 12.5 Gy/1fx	NR	NR	NR	NR	NR	GTV-[Median(range)/Gy]: D90 = 13.1 (11.7-17.9)

**Ennis et al., 2015 [63]**	14	NR	NR	NR	NR	5.44 (range: 2.37-7.40)	NR	Ultrasound	LDR brachytherapy (103Pd)	PTV = prostate + 2	PTV = 100%<br>GTV = 200%	31.5 (range: 24-52) months	NR	NR	NR	NR	No patients experienced biochemical recurrence after the median follow-up time.
**Schild et al., 2014 [64]**	78	NR	NR	NR	not 100 %	NR	Vmedian = 2.18	mpMRI (T2w + DWI + DCE)	IMRT OR VMAT	PTV = prostate + 3	Prostate = 77.4 Gy/43fx<br>GTV = 83 Gy/43fx<br>Involved SV = 75-77.4 Gy/43fx	36 (range: 4-57) months	NR	NR	NR	NR	3-year biochemical control rate = 92%<br>3-year rates of local control, distant control and survival were 98%, 95%, and 95%, respectively
**Aluwini et al., 2013 [65]**	50	60%	40%	0%	100 %	Mean PSA: 8.2 (1.3–16)	Vmean = 1.2 (range: 0.46–4.1)	MRI	Cyberknife	PTV = prostate + 3	PTV = 38 Gy in 4fx<br>GTV = up to 44 Gy (for 14 patients who detected IPLs)	[Mean]: 23 (9–47) months	23%(8 %)	14%(2 %)	16%	3%	GTV-[Mean(range)/Gy]:<br>Dmean = 47.8 (40.3–53.8)<br>BF = 0%
**Buwenge et al., 2020; Ippolito et al., 2012 [66]**	44	14%	41%	46%	100 %	6.11 (range: 2.28-36.00)	NR	MRI (T2w)	IMRT	PTV = (prostate + SV) + 1cm (8 mm posteriorly)<br>PTVboost = (GTV + 5mm) + 1cm (8 mm posteriorly)	PTV = 72 Gy/40fx<br>PTVboost = 80 Gy/40fx	120 (range: 25-150) months	27.3% (2.3%)	13.6% (4.5%)	13.6% (4.5%)	16.1% (2.3%)	bFFS: 95.3% (5 yr); 90.1% (10 yr)<br>OS: 95.5% (5 yr)/87.8% (10 yr);

Reference

1. Cambria, R., et al., *Ultrahypofractionated radiotherapy for localized prostate cancer with simultaneous boost to the dominant intraprostatic lesion: a plan comparison.* Tumori, 2021: p. 3008916211011667-3008916211011667.
2. Udrescu, C., et al., *Potential interest of developing an integrated boost dose escalation for stereotactic irradiation of primary prostate cancer.* Physica medica, 2013. **30**(3): p. 320-325.
3. Kim, Y.J., K.J. Yoon, and Y.S. Kim, *Simultaneous integrated boost with stereotactic radiotherapy for dominant intraprostatic lesion of localized prostate cancer: a dosimetric planning study.* Scientific reports, 2020. **10**(1): p. 14713-14713.
4. Tree, A., et al., *Prostate stereotactic body radiotherapy with simultaneous integrated boost: Which is the best planning method?* Radiation oncology (London, England), 2013. **8**(1): p. 228-228.
5. Murray, L.J.F., et al., *Prostate Stereotactic Ablative Radiation Therapy Using Volumetric Modulated Arc Therapy to Dominant Intraprostatic Lesions.* International journal of radiation oncology, biology, physics, 2014. **89**(2): p. 406-415.
6. Ashida, R., et al., *Highly hypofractionated intensity-modulated radiation therapy for nonmetastatic prostate cancer with a simultaneous integrated boost to intraprostatic lesions: a planning study.* Japanese journal of radiology, 2022. **40**(2): p. 210-218.
7. Ciabatti, S., et al., *Dominant intraprostatic lesion boosting in sexual-sparing radiotherapy of prostate cancer: A planning feasibility study.* Medical dosimetry : official journal of the American Association of Medical Dosimetrists, 2019. **44**(4): p. 356-364.
8. Azzeroni, R., et al., *Biological optimization of simultaneous boost on intra-prostatic lesions (DILs): Sensitivity to TCP parameters.* Physica medica, 2012. **29**(6): p. 592-598.
9. Amini, A., et al., *Dose painting to treat single-lobe prostate cancer with hypofractionated high-dose radiation using targeted external beam radiation: Is it feasible?* Medical Dosimetry, 2015. **40**(3): p. 256-261.
10. Thomas, L., et al., *68Ga-PSMA-PET/CT imaging of localized primary prostate cancer patients for intensity modulated radiation therapy treatment planning with integrated boost.* European journal of nuclear medicine and molecular imaging, 2018. **45**(7): p. 1170-1178.
11. Wang, T., et al., *A planning study of focal dose escalations to multiparametric MRI-defined dominant intraprostatic lesions in prostate proton radiation therapy.* British journal of radiology, 2020. **93**(1107): p. 20190845-20190845.
12. Maggio, A., et al., *Feasibility of safe ultra-high (EQD2>100 Gy) dose escalation on dominant intra-prostatic lesions (DILs) by Helical*

Tomotheraphy. Acta oncologica, 2011. **50**(1): p. 25-34.
13. Nutting, C., et al., *Potential improvements in the therapeutic ratio of prostate cancer irradiation: dose escalation of pathologically identified tumour nodules using intensity modulated radiotherapy.* The British journal of radiology, 2002. **75**(890): p. 151-161.
14. van Lin, E.N., et al., *IMRT boost dose planning on dominant intraprostatic lesions: gold marker-based three-dimensional fusion of CT with dynamic contrast-enhanced and 1H-spectroscopic MRI.* International Journal of Radiation Oncology* Biology* Physics, 2006. **65**(1): p. 291-303.
15. Zamboglou, C., et al., *Focal dose escalation for prostate cancer using 68Ga-HBED-CC PSMA PET/CT and MRI: A planning study based on histology reference.* Radiation oncology (London, England), 2018. **13**(1): p. 81-81.
16. Blake, S.W., et al., *A study of the clinical, treatment planning and dosimetric feasibility of dose painting in external beam radiotherapy of prostate cancer.* Physics and Imaging in Radiation Oncology, 2020. **15**: p. 66-71.
17. Pinkawa, M., et al., *Intensity-Modulated Radiotherapy for Prostate Cancer Implementing Molecular Imaging with 18F-Choline PET-CT to Define a Simultaneous Integrated Boost.* Strahlentherapie und Onkologie, 2010. **186**(11): p. 600-606.
18. Pinkawa, M., et al., *Dose-escalation using intensity-modulated radiotherapy for prostate cancer – Evaluation of the dose distribution with and without18 F-choline PET-CT detected simultaneous integrated boost.* Radiotherapy and oncology, 2009. **93**(2): p. 213-219.
19. Ost, P., et al., *Volumetric arc therapy and intensity-modulated radiotherapy for primary prostate radiotherapy with simultaneous integrated boost to intraprostatic lesion with 6 and 18 MV: a planning comparison study.* International Journal of Radiation Oncology* Biology* Physics, 2011. **79**(3): p. 920-926.
20. Bossart, E.L.P., et al., *Feasibility and Initial Dosimetric Findings for a Randomized Trial Using Dose-Painted Multiparametric Magnetic Resonance Imaging–Defined Targets in Prostate Cancer.* International journal of radiation oncology, biology, physics, 2016. **95**(2): p. 827-834.
21. De Meerleer, G., et al., *The magnetic resonance detected intraprostatic lesion in prostate cancer: planning and delivery of intensity-modulated radiotherapy.* Radiotherapy and oncology, 2005. **75**(3): p. 325-333.
22. Chang, J.H., et al., *Intensity Modulated Radiation Therapy Dose Painting for Localized Prostate Cancer Using 11C-choline Positron Emission Tomography Scans.* International journal of radiation oncology, biology, physics, 2012. **83**(5): p. e691-e696.
23. Kuang, Y.P., et al., *Volumetric Modulated Arc Therapy Planning for Primary Prostate Cancer With Selective Intraprostatic Boost Determined by18 F-Choline PET/CT.* International journal of radiation oncology, biology, physics, 2015. **91**(5): p. 1017-1025.

24. Seppälä, J., et al., *Carbon-11 acetate PET/CT based dose escalated IMRT in prostate cancer.* Radiotherapy and Oncology, 2009. **93**(2): p. 234-240.
25. Housri, N., et al., *Parameters favorable to intraprostatic radiation dose escalation in men with localized prostate cancer.* International Journal of Radiation Oncology* Biology* Physics, 2011. **80**(2): p. 614-620.
26. Abedi, I., et al., *Dosimetric and Radiobiological Evaluation of Multiparametric MRI-Guided Dose Painting in Radiotherapy of Prostate Cancer.* Journal of medical signals and sensors, 2017. **7**(2): p. 114-121.
27. Yeo, I., et al., *Passive proton therapy vs. IMRT planning study with focal boost for prostate cancer.* Radiation oncology (London, England), 2015. **10**(1): p. 213-213.
28. Kim, Y., et al., *Class solution in inverse planned HDR prostate brachytherapy for dose escalation of DIL defined by combined MRI/MRSI.* Radiotherapy and oncology, 2008. **88**(1): p. 148-155.
29. Pouliot, J., et al., *Inverse planning for HDR prostate brachytherapy used to boost dominant intraprostatic lesions defined by magnetic resonance spectroscopy imaging.* International Journal of Radiation Oncology* Biology* Physics, 2004. **59**(4): p. 1196-1207.
30. Crook, J., et al., *Ultrasound-planned high-dose-rate prostate brachytherapy: dose painting to the dominant intraprostatic lesion.* Brachytherapy, 2014. **13**(5): p. 433-441.
31. Kazi, A., et al., *MRS-guided HDR brachytherapy boost to the dominant intraprostatic lesion in high risk localised prostate cancer.* BMC cancer, 2010. **10**(1): p. 472-472.
32. DiBiase, S.J., et al., *Magnetic resonance spectroscopic imaging-guided brachytherapy for localized prostate cancer.* International Journal of Radiation Oncology* Biology* Physics, 2002. **52**(2): p. 429-438.
33. Mason, J., et al., *Multi-parametric MRI-guided focal tumor boost using HDR prostate brachytherapy: A feasibility study.* Brachytherapy, 2014. **13**(2): p. 137-145.
34. Dankulchai, P., et al., *Optimal source distribution for focal boosts using high dose rate (HDR) brachytherapy alone in prostate cancer.* Radiotherapy and oncology, 2014. **113**(1): p. 121-125.
35. Tree, A.C., et al., *Standard and Hypofractionated Dose Escalation to Intraprostatic Tumor Nodules in Localized Prostate Cancer: 5-Year Efficacy and Toxicity in the DELINEATE Trial.* International journal of radiation oncology, biology, physics, 2023. **115**(2): p. 305-316.
36. Eade, T., et al., *Early Outcomes and Decision Regret Using PSMA/MRI-Guided Focal Boost for Prostate Cancer SBRT.* Practical Radiation Oncology, 2022. **12**(3): p. e201-e206.

37. Strnad, V., et al., *Brachytherapy focal dose escalation using ultrasound based tissue characterization by patients with non-metastatic prostate cancer: Five-year results from single-center phase 2 trial.* Brachytherapy, 2022. **21**(4): p. 415-423.
38. Dankulchai, P., W. Sittiwong, and W. Teerasamit, *Feasibility and safety of definite volumetric modulated arc therapy with simultaneous integrated boost to the dominant intraprostatic lesion in patients with unfavorable intermediate to high-risk prostate cancer.* Reports of practical oncology and radiotherapy, 2022. **27**(2): p. 260-267.
39. Zamboglou, C., et al., *PSMA-PET-and MRI-Based Focal Dose Escalated Radiation Therapy of Primary Prostate Cancer: Planned Safety Analysis of a Nonrandomized 2-Armed Phase 2 Trial (ARO2020-01).* International Journal of Radiation Oncology* Biology* Physics, 2022. **113**(5): p. 1025-1035.
40. Hannan, R., et al., *SABR for high-risk prostate cancer: A prospective multilevel MRI-based dose escalation trial.* International Journal of Radiation Oncology* Biology* Physics, 2022. **113**(2): p. 290-301.
41. Kerkmeijer, L.G., et al., *Focal Boost to the Intraprostatic Tumor in External Beam Radiotherapy for Patients With Localized Prostate Cancer: Results From the FLAME Randomized Phase III Trial.* Journal of Clinical Oncology, 2021: p. JCO. 20.02873.
42. Zapatero, A., et al., *MRI-guided focal boost to dominant intraprostatic lesion using volumetric modulated arc therapy in prostate cancer. Results of a phase II trial.* British journal of radiology, 2021: p. 20210683-20210683.
43. Rezaeijo, S.M., et al., *The feasibility of a dose painting procedure to treat prostate cancer based on mpMR images and hierarchical clustering.* Radiation oncology (London, England), 2021. **16**(1): p. 1-182.
44. Kuisma, A., et al., *Long-term outcome of biologically guided dose-escalated radiotherapy of localized prostate cancer.* Acta oncologica, 2021: p. 1-7.
45. Armstrong, S., et al., *Single dose high-dose-rate brachytherapy with focal dose escalation for prostate cancer: Mature results of a phase 2 clinical trial.* Radiotherapy and oncology, 2021. **159**: p. 67-74.
46. Sanmamed, N., et al., *Tumor-targeted dose escalation for localized prostate cancer using MR-guided HDR brachytherapy (HDR) or integrated VMAT (IB-VMAT) boost: Dosimetry, toxicity and health related quality of life.* Radiotherapy and Oncology, 2020. **149**: p. 240-245.
47. Nicholls, L., et al., *Stereotactic radiotherapy with focal boost for intermediate and high-risk prostate cancer: Initial results of the SPARC trial.* Clinical and Translational Radiation Oncology, 2020. **25**: p. 88-93.
48. Murray, J.R., et al., *Standard and hypofractionated dose escalation to intraprostatic tumor nodules in localized prostate cancer: efficacy*

and toxicity in the DELINEATE trial. International Journal of Radiation Oncology* Biology* Physics, 2020. **106**(4): p. 715-724.
49. Marvaso, G., et al., *Phase II prospective trial "Give Me Five" short-term high precision radiotherapy for early prostate cancer with simultaneous boost to the dominant intraprostatic lesion: the impact of toxicity on quality of life (AIRC IG-13218).* Medical oncology (Northwood, London, England), 2020. **37**(8): p. 74-74.
50. Draulans, C., et al., *Primary endpoint analysis of the multicentre phase II hypo-FLAME trial for intermediate and high risk prostate cancer.* Radiotherapy and Oncology, 2020. **147**: p. 92-98.
51. Alayed, Y., et al., *Evaluating the tolerability of a simultaneous focal boost to the gross tumor in prostate SABR: a toxicity and quality-of-life comparison of two prospective trials.* International Journal of Radiation Oncology* Biology* Physics, 2020. **107**(1): p. 136-142.
52. Herrera, F.G., et al., *50-Gy stereotactic body radiation therapy to the dominant intraprostatic nodule: results from a phase 1a/b trial.* International Journal of Radiation Oncology* Biology* Physics, 2019. **103**(2): p. 320-334.
53. McDonald, A.M., et al., *Prostate stereotactic body radiation therapy with a focal simultaneous integrated boost: acute toxicity and dosimetry results from a prospective trial.* Advances in radiation oncology, 2019. **4**(1): p. 90-95.
54. Guimond, E., et al., *Impact of a dominant intraprostatic lesion (DIL) boost defined by sextant biopsy in permanent I-125 prostate implants on biochemical disease free survival (bDFS) and toxicity outcomes.* Radiotherapy and oncology, 2019. **133**: p. 62-67.
55. Alayed, Y., et al., *MRI assisted focal boost integrated with HDR monotherapy study in low and intermediate risk prostate cancer (MARS): Results from a phase II clinical trial.* Radiotherapy and oncology, 2019. **141**: p. 144-148.
56. Timon, G., et al., *Short-term high precision radiotherapy for early prostate cancer with concomitant boost to the dominant lesion: Ad interim analysis and preliminary results of Phase II trial AIRC-IG-13218.* British journal of radiology, 2018. **91**(1089): p. 20160725-20160725.
57. Schlenter, M., et al., *Intensity-modulated radiotherapy of prostate cancer with simultaneous integrated boost after molecular imaging with 18F-choline-PET/CT: Clinical results and quality of life.* Strahlentherapie und Onkologie, 2018. **194**(7): p. 638-645.
58. Onjukka, E., et al., *Twenty fraction prostate radiotherapy with intra-prostatic boost: results of a pilot study.* Clinical Oncology, 2017. **29**(1): p. 6-14.
59. Uzan, J., A. Nahum, and I. Syndikus, *Prostate dose-painting radiotherapy and radiobiological guided optimisation enhances the therapeutic ratio.* Clinical Oncology, 2016. **28**(3): p. 165-170.
60. Sundahl, N., et al., *Combining high dose external beam radiotherapy with a simultaneous integrated boost to the dominant intraprostatic lesion: Analysis of genito-urinary and rectal toxicityToxicity after integrated boost for prostate cancer.* Radiotherapy and oncology, 2016.

119(3): p. 398-404.
61. King, M.T., et al., *Long-term outcome of magnetic resonance spectroscopic image–directed dose escalation for prostate brachytherapy.* Brachytherapy, 2016. **15**(3): p. 266-273.
62. Gomez-Iturriaga, A., et al., *Dose escalation to dominant intraprostatic lesions with MRI-transrectal ultrasound fusion High-Dose-Rate prostate brachytherapy. Prospective phase II trial.* Radiotherapy and oncology, 2016. **119**(1): p. 91-96.
63. Ennis, R.D., et al., *Phase I/II prospective trial of cancer-specific imaging using ultrasound spectrum analysis tissue-type imaging to guide dose-painting prostate brachytherapy.* Brachytherapy, 2015. **14**(6): p. 801-808.
64. Schild, M.H., et al., *Early outcome of prostate intensity modulated radiation therapy (IMRT) incorporating a simultaneous intra-prostatic MRI directed boost.* OMICS journal of radiology, 2014. **3**(4).
65. Aluwini, S., et al., *Stereotactic body radiotherapy with a focal boost to the MRI-visible tumor as monotherapy for low-and intermediate-risk prostate cancer: early results.* Radiation Oncology, 2013. **8**(1): p. 1-7.
66. Buwenge, M., et al., *Simultaneous integrated radiotherapy boost to the dominant intraprostatic lesion: final results of a phase I/II trial.* Anticancer Research, 2020. **40**(11): p. 6499-6503.
